# Health related social exclusion in Europe: a multilevel study of the role of welfare generosity

**DOI:** 10.1186/1475-9276-12-81

**Published:** 2013-09-28

**Authors:** Therese Saltkjel, Espen Dahl, Kjetil A van der Wel

**Affiliations:** 1 Department of Social Work, Child Welfare and Social Policy, Faculty of Social Sciences, Oslo and Akershus University College of Applied Sciences, Post Box 4, St Olavs Plass N-0130 Oslo, Norway

**Keywords:** Health, Social position, Social participation, Social exclusion, Welfare state generosity

## Abstract

**Introduction:**

The aim of this paper was to investigate the association between health, social position, social participation and the welfare state. Extending recent research on the social consequences of poor health, we asked whether and how welfare generosity is related to the risk of social exclusion associated with combinations of poor health, low education and economic inactivity.

**Methods:**

Our analyses are based on data from the European Social Survey, round 3 (2006/7), comprising between 21,205 and 21,397 individuals, aged 25–59 years, within 21 European welfare states. The analyses were conducted by means of multilevel logistic regression analysis in STATA 12.

**Results:**

The results demonstrated that the risk of non-participation in social networks decreased as welfare generosity increased. The risk of social exclusion, i.e. non-participation in social networks among disadvantaged groups, seldom differed from the overall association, and in absolute terms it was invariably smaller in more generous welfare state contexts.

**Conclusions:**

The results showed that there were no indications of higher levels of non-participation among disadvantaged groups in more generous welfare states. On the contrary, resources made available by the welfare state seemed to matter to all individuals in terms of overall lower levels of non-participation. As such, these results demonstrate the importance of linking health related social exclusion to the social policy context.

## Introduction

Poor health might have social and economic consequences [1,2]. Comparative health inequality research has recently directed attention in particular to the opportunities to participate in the labour market, and how they relate to the social policy context [3-7]. These studies demonstrate higher employment rates among individuals in poor health in more comprehensive welfare states. Extending this emerging field of research beyond employment studies, we ask whether and how welfare generosity is related to the risk of social exclusion in terms of non-participation associated with combinations of poor health, low education and economic inactivity. As such our research adds to the existing literature an empirical extension of the social consequences of illness-concept. We also use insights from the concepts of social capital and social exclusion on how and why social networks and social participation are important dimensions of full participation and welfare in society. Thus, the paper also adds new knowledge to the social participation and social exclusion literature by explicitly studying social inequality in the association between welfare state arrangements and social participation.

There are at least two contradictory perspectives on the role of welfare states in the formation of social participation. One emphasizes the role of collective resources made available by the welfare state in enabling and stimulating social participation, what we label the *welfare resources perspective*[8,9]. The other perspective, the *crowding-out hypothesis*, warns against detrimental effects of large welfare states: they cause deterioration in civic engagement by taking over tasks traditionally carried out by families and social networks [10,11]. The aim of this paper is to further investigate these two contrasting hypotheses.

## Theoretical considerations

The concepts of social exclusion and social capital emphasize the importance of participation in social networks [12]. According to the concept of *social exclusion*, individuals who lack or are denied access to full and active participation in all or at least key aspects of customary social life are at risk of social exclusion [12,13]. Social networks and civic participation are two important dimensions of social exclusion, among a number of other dimensions [14]. Within the conceptual framework of social exclusion, social relations provide social support and prevent social isolation [12]. The concept of *social capital*, although acknowledging these potential gains, has much broader expectations of the benefits of social networks: they may also provide material resources, practical assistance, information, and so on. The result of participation in networks might thus be the ability to achieve objectives otherwise not available to the individual on his/her own [15].

Putnam [16] offers an applicable distinction between two different forms of networks, that is, 'bonding’ and 'bridging’ networks. While bonding networks tend to be inward looking, maintaining homogeneity, bridging networks are heterogeneous and outward-looking and link people across social divides, with the potential of accessing resources beyond their own bonding networks [16,17]. Taken together, social participation gives access to resources important to both 'get by’ and to 'get ahead’ [16,18]. The greater the command over resources, the greater the leeway to realize one’s life chances [19].

In the social capital literature, social participation appears to be the antidote to social exclusion [12]. In Putnam’s somewhat communitarian approach, social capital is non-exclusive and accessible to all [15,16]. Disadvantaged individuals need only to participate and enjoy the payoffs. Putnam’s approach ultimately implies that redistribution is unnecessary [12,16]. However, the communitarian approach to social capital is contested. Lin [20] argues that social capital is unequally distributed across social positions, because individuals tend to associate with others of similar socio-economic characteristics. Hence, not all networks have equal amounts of resources.

Poor health, low social position and economic inactivity, although sometimes regarded as part of the social exclusion phenomenon, are also important risk factors of social exclusion [14]. Numerous empirical studies have demonstrated the association between these risks and social participation [10,16,21-28]. Individuals in poor health may be excluded from social participation in several ways. First, poor health may independently hinder social participation because of lack of physical or psychological energy necessary to interact with other people. Poor health may also be accompanied by physical impairments, which could make the ill person withdraw in shame, or suffer from the discrimination of others. Second, when combined with low educational level or non-employment, the risk of social isolation and exclusion increases because of the deprivation of important financial resources, human capital, and work-related social networks. Third, as pointed out by Lin [20], the quality, in terms of resources, of the social networks which are available to disadvantaged groups may be substantially poorer, or even detrimental to certain outcomes, and may hinder participation in bridging social networks. Hence, social disadvantage may be reproduced in bonding social networks through 'vicious and virtuous circles’ [29], and increase the risk of social exclusion.

We theoretically assume that participation in different networks is an essential part of customary social life and hence one important dimension of social exclusion. Although there are also other important dimensions, in this paper we study participation, or rather *non* –participation, and refer to it as *risk of* social exclusion.

## The social policy context

The role of welfare states in the formation of social participation is a matter of dispute [10,11,16,24,30-33]. The *welfare resources perspective* hypothesizes that generous welfare states may buffer the extent to which social disadvantage in one area of life causes disadvantage in another area of life, and hence diminish the risk of cumulative disadvantage and social exclusion. For instance, labour market exclusion may or may not lead to a weakening of social ties, depending on the level of freedom experienced by the non-employed in terms of participatory resources: being able to receive guests at home or bring a small gift to a party; having proper clothing to attend social events; having the financial resources to travel, or to pay participation fees in voluntary organizations, and so on. The more generous social benefits are, the less likely it is that job loss will lead to social exclusion. Similarly, welfare resources can enable individuals in poor health to overcome health impairments that would otherwise hinder social participation, for example, having the financial resources to buy medicine or having access to publicly funded/subsidized aids (e.g. wheelchair, hearing aid, etc.), or personal assistance. As for the low educated, having the financial resources to attend sports, leisure or cultural activities might give access to diverse social arenas and bridging networks otherwise not available through one’s own network of similar socio-economic characteristics [20].

Participatory resources may be individual (e.g. savings, skills, health, etc.) or provided by family, or they may be collective, for example, provided by the welfare state. From a welfare resources perspective [8] we would expect lower levels of non-participation among disadvantaged groups in more generous welfare states, because more resources are made available to them.

On the other hand, according to traditional Anglo-American state theory, a strong state undermines civil society [34]. This perspective, the crowding-out hypothesis, suggests that generous welfare states mute formation of social networks, social relations and civic participation through colonizing tasks otherwise tended by families and local communities [11]. This 'crowding out’ of civic society may lead to increased welfare dependency, weakening people’s ability to work with one another and cooperate and their willingness to participate and engage in public affairs, resulting in increased social isolation, weakening of moral ties and anomie [10,11,33]. Social networks and the supportiveness of local social relations available to disadvantaged groups in generous welfare states may therefore be insufficient to prevent and alleviate social exclusion. For instance, someone living in a generous welfare state, who loses her job, may have sufficient income, due to generous benefits, but may still have an elevated risk of social exclusion compared to the corresponding case in a moderate welfare state. This is because the quality and extent of social networks, communities and voluntary organizations is less evolved, and hence they are less able to include the unemployed person in meaningful activities and provide social support, resulting in an increased risk of cumulative disadvantage and social exclusion. Thus, the crowding-out hypothesis expects higher levels of non-participation among disadvantaged groups in more generous welfare states.

## Welfare generosity and social participation – previous findings

The majority of recent studies demonstrated that welfare state matters in terms of (average levels of) social capital, including formal and informal social participation among the citizens [35]. Some of the most recent (mainly) multilevel comparative studies nonetheless showed that the results in some instances depend on the measure of social participation applied (formal vs. informal). In addition, the scarce results on the impact of welfare generosity among disadvantaged groups appear inconclusive. Finally, most of the studies applied variations of a welfare generosity measure based on social expenditure as percentage of GDP, none of which took the extent of 'need’ in the population into account [36].

Gesthuizen et al. [24] showed that the national level of social security did not affect informal social capital, but increased membership in voluntary organizations [24]; the latter was also demonstrated in a former study by Gesthuizen et al. [37]. Anderson [21] demonstrated that individuals in countries with higher spending on active labour market policies reported more frequent social interactions, increased membership in voluntary organizations and a reduced sense of social exclusion. Moreover, the positive associations were found for both labour-market insiders and outsiders; however, the association with social ties and perception of social inclusion were stronger among outsiders (unemployed actively/not actively looking) [21].

Gelissen et al. [35] demonstrated that welfare generosity was associated with most of the individual-level factors, including participation in formal networks, which tended to be significantly higher among individuals who live in countries with higher levels of welfare provision. There was, however, no significant effect on contact frequency with friends by welfare generosity [35]. Van Ingen and van der Meer [38] also demonstrated that inequality in organizational participation across education, gender and income were smaller in countries with higher levels of welfare generosity. In the study by van der Meer et al. [39] the results showed that welfare generosity did not have a significant effect on participation within the nuclear family, or on interaction with friends. However, welfare generosity had a crowding-out effect on participation within the extended family (uncle, aunt, cousin) [39]. Moreover, this negative effect was stronger for people with a low income than for people with a high income. The study nonetheless demonstrated a significant and positive effect of welfare generosity on social participation with nuclear and extended family among disabled individuals. Another multilevel study provided support for the crowding-out thesis in that volunteering was lower in extensive welfare states than in countries that spent less on welfare state policy; however, welfare generosity did not significantly affect volunteering among low-income groups [31]. Finally, the comparative study by van Oorschot and Finsveen [29] showed no clear relationship between lower inequality of social capital and more developed welfare states.

To sum up, the variables measuring participation in formal and informal networks varied between the reviewed studies. In the literature formal networks were often exemplified by contacts within voluntary organizations. Friends, family, neighbours and colleagues were common examples of informal networks [17]. The distinction between bridging and bonding networks within the conceptual framework of social capital is related to the social characteristics of the members in the networks. In the former case the networks are heterogeneous, and in the latter homogeneous, in terms of social characteristics [17]. Despite these conceptual differences, both informal networks and (strong) bonds provide emotional support, while both formal networks and (weak) bridges provide (wide) formal support [17].

It seems that most of the variables on participation in informal networks in the studies reviewed measured frequencies of contact with informal networks. In the studies highlighted here it was only the study by Anderson [21] that demonstrated a significant positive effect of welfare generosity on informal networks. The study by van der Meer et al. [39] also showed a significant negative effect on participation with extended family, while a significant positive effect among disabled individuals on social participation with nuclear and extended family. Most of these studies nonetheless demonstrated a significant positive effect of welfare generosity on participation in formal networks, with the exception of the study by Stadelman-Steffen [31], who demonstrated a significant negative effect among upper and middle social classes. Due to these rather inconsistent findings, in this study we chose to apply two dependent variables, that is, *non-participation* in *formal* and *informal* networks, within the context of the available data of the European Social Survey (2006/7).

Social expenditure as percentage of GDP is one of the most commonly used indicator of welfare generosity in the empirical literature [31]. The expenses approach has been criticized for not sufficiently addressing social citizenship and social rights as core defining characteristics of the welfare state [40]. An institutional approach however was not possible in this paper as available comparative databases (SCIP and CWED) only comprise about half of the countries (12 out of 21) included in our analyses [40-42]. The validity of the institutional approach has also met objections for the underlying assumption of the 'average production worker’ [43]; a description which does not fit many Europeans today [40]. Another main criticism of the expenses, or welfare generosity approach, is that welfare state effort becomes relative to the size of the GDP, while what matters to people is the level of living that social spending buys [36]. Furthermore, higher social expenditures may only reflect higher social needs, such as mass unemployment, and may not reflect adequately the average resources made available to people not provided for by the market. Therefore, a measure of welfare generosity should take the extent of 'need’ in the population into account [36]. The present paper meets these objections in that we used a measure of social expenditure in purchasing power standard (PPS) per capita inhabitant, adjusted for the level of need in each country (see also [44]). As far as we know, this is the first study of welfare state generosity and social participation that applies social spending data in this way. In addition, this study adds to the existing knowledge on group-specific effects of welfare generosity [31], including groups with double-disadvantages that is, poor health combined with either low education or non-employment. Finally, this study extends previous research on the social consequences of poor health within a welfare state context, including social participation as an outcome.

## Data and methods

### The data set

This article is based on the repeat cross-sectional *European Social Survey* (ESS), round 3 (2006/7) [45]. The overall aim of the ESS survey is to monitor public attitudes and values and to study how these change and interact with institutions within Europe [46]. The ESS3 integrated file net sample size is 43,000 individuals within 23 countries. For more information about sample size, response rates, and so on, see the ESS Documentation Report [46].

Our analyses included between 21,205 and 21,397 respondents, aged between 25 and 59 years, living in 21 European countries (see Table 1). Ukraine and Russia were left out of the analysis, due to missing data on the welfare generosity measure.

**Table 1:** Welfare generosity and proportions of individuals (aged 25–59 years) within countries reporting non-participation in formal and informal networks, poor health, non-employment and educational attainment (%)

**Country**	**Welfare generosity**	**Non-participation – formal**	**Non-participation – informal**	**Poor health**	**Non-employed**	**Primary**	**Secondary**	**Tertiary**
Norway	220.75	34.6	5.69	4.27	12.61	9.02	33.14	57.83
Denmark	180.83	39.21	8.66	3.58	14.72	12.87	34.62	52.51
Netherland	176.06	49.93	6.88	3.25	28.11	32.6	27.75	39.65
Switzerland	173.94	36.65	7.77	2.14	23.59	17.42	48.4	34.18
Sweden	162.66	58.52	10.06	3.35	9.6	33.27	31.5	35.23
United Kingdom	127.31	49.91	20.81	4.55	20.77	37.18	12.23	50.59
Finland	113.82	59.7	12.82	2.39	15.71	16.0	41.15	42.84
Austria	112.34	42.14	11.68	2.69	20.49	13.52	66.9	19.57
Germany	109.66	49.92	17.78	6.93	27.29	7.08	58.03	34.9
Ireland	108.63	44.32	25.11	2.61	30.99	28.72	22.69	48.59
France	96.44	49.73	12.72	5.22	20.63	19.95	47.22	32.83
Belgium	93.69	50.3	13.08	3.17	24.36	21.88	40.1	38.02
Spain	72.81	56.03	8.84	4.92	21.75	42.47	19.39	38.14
Slovenia	63.11	52.98	28.55	6.17	27.14	16.83	56.3	26.88
Portugal	61.29	76.36	4.94	8.34	24.82	68.17	17.86	13.97
Cyprus	55.70	75.9	27.92	2.14	28.97	19.27	52.34	28.39
Hungary	34.43	80.43	47.66	11.41	31.31	23.45	53.47	23.08
Slovakia^b^	26.84	74.4	19.71	5.78	26.44	10.28	75.42	14.3
Estonia	24.95	78.8	23.75	5.69	15.42	10.24	44.75	45.01
Poland	17.88	81.67	40.41	8.27	33.27	15.24	64.36	20.4
Bulgaria	11.99	89.23	22.66	9.31	33.46	23.1	51.57	25.33

*Source*: ESS3 2006/7 [45]. A design weight (dweight) has been applied in the ESS dataset to correct for different probabilities of selection.

*Source*: Eurostat, ESSPROS (welfare generosity) [47].

^b^Due to lack of social spending data on housing and social exclusion for Slovakia for the year 2006, we have included the mean of the years 2002–2004.

### Dependent variables

Two dependent variables were included in this study in order to measure two important dimensions of social exclusion, that is, non-participation in informal and formal networks. Non-participation in informal networks was assessed by the following question: 'How often do you meet socially with friends/relatives/ work colleagues?’. The answers within the range of 1 'never’ to 7 'every day’, were dichotomized such that the responses 'never’, 'less than once a month’ and 'once a month’ were given the value of 1, labelled 'non-participation in informal networks’. All the other values were given the value 0.

Non-participation in formal networks is a measure including two questions, with responses ranging from 1 to 6: (a) 'In the past 12 months, how often did you get involved in work for voluntary or charitable organizations?’ and (b) 'In the past 12 months, how often did you help with or attend activities in your local area?’. The response options ranged from 1 'at least once a week’ to 6 'never’. The two variables were collapsed and dichotomized. The responses for both variables, including 'never’ and 'less often’ ('than at least once every six months’) were given the value of 1, labelled 'non-participation in formal networks’. All the other responses were given the value 0.

### Contextual variable

The contextual variable labelled 'welfare generosity’ was measured in purchasing power standards per capita, including social protection benefits (direct transfers in cash or in kind) on unemployment, sickness and disability, housing, and social exclusion, derived from the Eurostat database, *The European System of Integrated Social Protection Statistics* (ESSPROS), for the year 2006 [47]. The sum of the social protection benefits was divided by the inverse of Eurostat’s employment rate in the age group 15-64 years for the year 2006. The aim was, although imperfectly, to adjust for the level of need in each country [36,44]. This measure has previously been applied by van der Wel et al. [6,40].

### Independent variables

We computed two sets of combined dummy variables to measure social disadvantage. One (set 1) consisted of combinations of the indicator variables, non-employment and self-perceived health. The other (set 2), included combinations of educational attainment and self-perceived health. This approach resulted in 4 group variables in set 1, and 6 group variables in set 2.

Self-perceived health was assessed by the question 'How is your (physical and mental) health in general?’ The response options were 'very good’, 'good’, 'fair’, 'bad’ and 'very bad’. The variable was dichotomized such that the response options 'very good’, 'good’, and 'fair’ were given the value 0. The responses 'bad’ and 'very bad’ were given the value 1, labelled 'poor health’.

Non-employment was assessed by a question measuring the respondent’s main activity during the previous 7 days. Nine response categories were eligible. The variable applied was the post-coded version of this variable available in the data file [48]. The categories 'in paid work’, 'in education’ and 'in community service’ were included in the category labelled 'employed’ (0). All the other categories, that is, 'unemployed’, 'permanently sick or disabled’, 'retired’, 'doing housework’, 'looking after children’ and 'other’ were included in the category labelled 'non-employed’ (1). Educational attainment was based on a harmonized variable based on country specific questionnaire item(s) assessed by the question: 'What is the highest level of education you have achieved?’ The coding is based on the *International Standard Classification of Education* (ISCED-1997) [49]. The variable was recoded into three dummy variables: 'primary’ (ISCED 0–1 and 2), 'secondary’ (ISCED 3) and 'tertiary’ education (ISCED 4 and 5–6).

As control variables we included gender, age, living with children, immigration status (born in the country), and marital status. Marital status was coded as 'married or cohabiting’ (value 1) for responses 'married’ and 'in civil partnership’. All other responses 'separated (still legally married)’, 'separated (still in a civil partnership)’, 'divorced’, 'widowed’, 'formerly in civil partnership, now dissolved’, 'formerly in civil partnership, partner died’, 'never married AND never in a civil partnership’ were given the value 0.

### Analysis – multilevel approach

The underlying assumption of multilevel models is that individuals are shaped by the social context to which they belong, that is, individuals from the same countries are more alike than individuals from different countries [50]. Thus, the advantage of multilevel techniques in the setting of the present paper is the opportunity to simultaneously study the effects of individual-level variables, contextual variables and cross-level influences on an individual-level outcome. We used multilevel random intercept analysis with binary outcomes available in the xtlogit procedure in STATA 12. A design weight was applied in the descriptive analyses to correct for different probabilities of selection [46]. The multilevel analyses were not weighted. In the random intercept model, a significant intercept variance indicates a systematic variation in the outcome variable from country to country (i.e. random effect). However, within the countries the effect of explanatory variables applies to all cases [50].

Our analytical approach was to analyse the impact of different constellations of poor health combined with other social disadvantages on social participation. Therefore, for each of the two dependent variables, two separate analyses were carried out. One analysis included on the right-hand side the various possible health and employment combinations (set 1), and the other analysis included the education and health combinations (set 2). To assess whether the link between these different forms of social disadvantages and exclusion from social participation varied with welfare generosity, we used cross-level interaction terms.

## Results

### Descriptives

Table 1 shows the proportion in each country reporting poor health, non-employment and various levels of education, and who are classified as excluded from participation in informal and formal social networks. In the first column the level of welfare generosity in each country is also reported. The Eastern European countries had very high proportions of residents not participating in either informal or formal social networks, with Hungary as an extreme case (non-participation rates of 80.43% in formal networks and 47.66% informal networks). Within these countries the levels of welfare generosity were also the lowest. At the other end of the distribution within the Scandinavian countries, in particular, Denmark and Norway, the levels of welfare generosity were among the highest. Among the southern countries the results demonstrated that Portugal had the lowest proportion of non-participation in informal networks (4.9%) among all the countries in the study. However, Portugal had high proportions of non-participation in formal networks (76.36%). The Bismarckian countries seemed to take a middle position; however, there were variations. Switzerland had the lowest level of non-participation in formal networks in the study (36.65%), and low levels of non-participation in informal networks (7.77%). Lastly, within the Anglo-Saxon countries, the United Kingdom and Ireland, the proportions of non-participation in informal networks were rather high, 20.81% in the UK and 25.11% in Ireland, respectively.

### Multilevel analyses

Table 2 shows the analyses including each of the two dependent variables, i.e non-participation in informal (A) and formal (B) networks, and the various health and employment combinations (set 1). The results indicate that the risk of being excluded from participation in *informal* social networks (A) increased with age and lower educational level, and was higher among immigrants and among those who lived with children or were married/cohabiting. In addition, there was a significant difference between men and women in non-participation in informal networks, whereby men faced a lower risk (Table 2(A)).

**Table 2:** **Multilevel logistic regression analysis of non-participation in networks: informal (A) and formal (B), *self-rated health and employment*, and welfare generosity, unstandardized regression coefficients (B), (N = 21,397 and 21,205 individuals nested within N = 21 countries)**

**Variables**	**Informal (A)**	**Formal (B)**
*Fixed part*		
Intercept	-2.494***	2.239***
Gender (male =1, female = 0)	-.08578*	-.07049*
Age	.03039***	-.00815***
Education (ref. Tertiary)		
Primary	.5656***	.7662***
Secondary	.312***	.4133***
Children (1 = yes, 0 = no)	.3082***	-.321***
Born in the country (1 = yes, 0 = no)	-.2116**	-.4721***
Married or cohabiting	.1033*	-.2446***
Health and employment (ref. Good health and employed)		
Good health and non-employed	-.03874	.2165*
Poor health and employed	-.07949	.1042
Poor health and non-employed	.3039	.3233
Contextual variable		
Welfare generosity	-.009018***	-.01058***
Cross-level interaction terms (ref. Good health and employed × welfare generosity)		
Good health and non-employed × welfare generosity	.000584	-.001479*
Poor health and employed × welfare generosity	.005266*	-.00097
Poor health and non-employed × welfare generosity	.003441*	-.00015
*Random part*		
Standard deviation of random intercept^b^	.591919	.385481
		
Intraclass correlation (ρ)	.096249	.043216
Log likelihood	-8597	-13143

*p < 0.05; **p < 0.01; ***p < 0.001.

^b^ all higher-level variances are significant at p < 0.001 (likelihood ratio test).

Null-model: non-participation, *informal*:

Standard deviation of random intercept^b^: 0.770282.

Intraclass correlation (ρ): 0.152795.

Null model: non-participation, *formal*:

Standard deviation of random intercept^b^: 0.735715.

Intraclass correlation (ρ): 0.141283.

Source: ESS3 2006/7 [45].

Source: Eurostat, ESSPROS (welfare generosity) [47].

The analysis including non-participation in *formal* networks (B) as dependent variable shows that the risk of non-participation increased with lower level of education and was higher among immigrants. The risk of non-participation in formal networks was however, lower among those who lived with children in the household and were married or cohabiting, and decreased with age. Again, men faced a lower risk than women.

Running the analysis without any contextual or interaction terms showed that the risk of non-participation increased with social disadvantage (results not reported). In the analysis including non-participation in *formal* networks (B) it was only the coefficient for those who reported poor self-rated health and were non-employed that was significant. However, due to the fixed level 1 residual variance (π^2^/3), we have to be cautious comparing regression coefficients and variances across models [50,51].

Column 1 in Table 2(A) shows that welfare generosity generally decreased the risk of non-participation in *informal* social networks. Due to the inclusion of cross-level interaction terms between the group variables and welfare generosity, the main effect of welfare generosity reflects the effect in the reference category, that is, those who are employed and in good health. The coefficient, however, also must be taken into account when evaluating the interaction terms. In a model without cross-level interaction terms the coefficient for welfare generosity was practically similar (not shown). Again, we have to be cautious comparing regression coefficients across models [51]. In Table 2(A) the results demonstrate no significant modifying effect of welfare generosity on non-participation in informal networks among disadvantaged individuals, that is, those who reported poor self-rated health and were non-employed.

The findings for non-participation in *formal* networks in the second column of Table 2(B) again exhibits strong negative effects of welfare generosity and few differences in the effect across social groups. However, a significant effect of welfare generosity could be observed for the category that reported good health and were non-employed (Table 2(B)).

Table 3 shows the analyses including each of the two dependent variables, i.e non-participation in informal (A) and formal (B) networks and the various health and education combinations (set 2). The results in Table 3(A) indicate that the risk of being excluded from participation in *informal* social networks increased with age, and was higher among immigrants and among those who lived with children or were married/cohabiting. The analysis including non-participation in *formal* networks as dependent variable (Table 3B) shows that the risk of non-participation in formal networks was higher among the non-employed, however lower among those who lived with children in the household and were married or cohabiting, and decreased with age. There was a significant difference between men and women, whereby men faced a lower risk. In models with no contextual variables the coefficients for the group variables indicated increasing risk of social exclusion with increasing disadvantage (tables not shown).

**Table 3:** **Multilevel logistic regression analysis of non-participation in networks: informal (A) and formal (B), *self-rated health and education, *and welfare generosity, unstandardized regression coefficients (B), (N = 21,397 and 21,205 individuals nested within N = 21 countries)**

**Variables**	**Informal (A)**	**Formal (B)**
*Fixed part*		
Intercept	-2.38***	2.178***
Gender (male = 1, female = 0)	-.07761	-.06898*
Age	.03031***	-.008031***
Children (1 = yes, 0 = no)	.3091***	-.3248***
Born in the country (1 = yes, 0 = no)	-.2112**	-.4658***
Married or cohabiting	.1036*	-.2459***
Non-employed	.03918	.07852*
Health and education (ref. Good health and tertiary)		
Poor health and tertiary	.4272	.4806
Good health and secondary	.1481	.5673***
Poor health and secondary	.2953	.5386*
Good health and primary	.315**	.8131***
Poor health and primary	.52*	.9887***
Contextual variable		
Welfare generosity	-.01037***	-.01017***
Cross-level interaction terms (ref. Good health and tertiary × welfare generosity)		
Poor health and tertiary × welfare generosity	.003356	-.00269
Good health and secondary × welfare generosity	.001865*	-.001372*
Poor health and secondary × welfare generosity	.006244**	-.0004
Good health and primary × welfare generosity	.002843*	-.00043
Poor health and primary × welfare generosity	.005119*	-.00008
*Random part*		
Standard deviation of random intercept^b^	.588563	.385413
		
Intraclass correlation (ρ)	.095264	.043201
Log likelihood	-8594	-13144

*p < 0.05; **p < 0.01; ***p < 0.001.

^b^ all higher level variances are significant at p < 0.001 (likelihood ratio test).

Null-model: non-participation, *informal*:

Standard deviation of random intercept^b^: 0.770282.

Intraclass correlation (ρ): 0.152795.

Null-model: non-participation, *formal*:

Standard deviation of random intercept^b^: 0.735715.

Intraclass correlation (ρ): 0.141283.

Source: ESS3 2006/7 [45].

Source: Eurostat, ESSPROS (welfare generosity) [47].

In Table 3(A) the coefficients for the cross-level interaction terms indicate a significant effect of welfare generosity on non-participation in *informal* networks for the most disadvantaged group, that is, those who reported poor self-rated health and primary education. However, the significant modifying effect for the group that report good health and primary education was somewhat stronger. Both coefficients were positive and smaller in strength than the main effect of welfare generosity, meaning that the combined effects of welfare generosity in these groups were attenuated compared to the most advantaged group.

**Figure 1:**
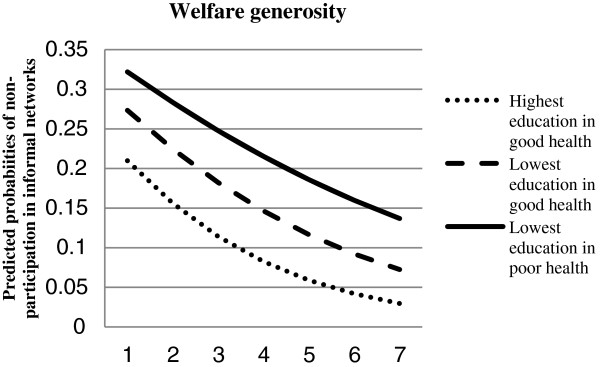
**Predicted probabilities of non-participation in informal networks by self-rated health and educational level within the actual observed range of values on welfare generosity.** Based on Table 3(A).

Figure 1 illustrates the effect of welfare generosity on non-participation in *informal* networks in predicted probabilities for an average individual (who is married, born in the country, and aged 42.4 years) within the actual observed range of values on welfare generosity. The first point along the horizontal axis (1) refers to the lowest observed value on welfare generosity (11.99). The second point (2) adds to the lowest observed value, the difference between the highest observed value (220.75) and the lowest (11.99), divided by 6 (11.99+34.79). At each point (3-6) 34.79 is added to the preceding sum (11.99+34.79+34.79 etc.). The shapes of the lines are slightly curvilinear with a deflection at high values of the welfare-generosity variable, due to the shape of the logistical curve (s-shape) [52]. The results demonstrate that the mean difference in effect between individuals who reported poor self-rated health and had the lowest level of education and individuals who reported good self-rated health and had the highest level of education was substantial.

The findings for non-participation in *formal* networks in the second column of Table 3(B) again exhibit strong negative effects of welfare generosity. Although there are few differences in the effect across social groups, a significant effect of welfare generosity could be observed for the category having good health and secondary education (Table 3(B)).

But how strong are the effects of welfare generosity on social participation? Calculations showed that the maximum effect of welfare generosity on non-participation in *informal* networks among the highest educated in good health (who were born in the country, married and aged 42 years), was 0.18. The maximum effect was calculated by subtracting the predicted group probability for the highest observed value on welfare generosity from the lowest observed value. Among the lowest educated in poor health the maximum effect was 0.19. Among the lowest educated in good health the maximum effect was 0.20. Compared to significant individual-level effects, expressed in predicted probabilities, the maximum effect of welfare generosity was as strong as, and stronger than most, other observed effects (e.g. the probability of not participating in informal social activities was 0.05 points higher for those living with children compared to those not living with children (0.06 points in a model including the cross-level interaction terms), 0.02 points higher for those who were married/cohabiting compared to those who were not and 0.20 points higher for those with poor health and low education compared to the most advantaged group. Hence, the effects found in this paper are quite substantial.

## Discussion

The aim of this paper was to study whether and how welfare generosity is related to the risk of social exclusion in terms of non-participation, associated with combinations of poor health, low education and economic inactivity. The results from our multilevel analysis of 21 European countries in the European Social Survey (2006/2007) demonstrated that welfare generosity decreased the risk of social exclusion in the face of poor health in combination with the risk factors of low educational attainment and non-employment, at least in absolute terms (i.e. the combined effect of the main effect of welfare generosity and its group specific effects, see Figure 1). The effects of welfare generosity on exclusion from formal and informal activities seldom differed from the main effect across all countries, indicating that all social groups benefitted equally from welfare generosity in terms of social participation. The strongest group differences were found in the model analysing the effect of welfare generosity for combinations of health and education on non-participation in *informal* networks, where relative inequalities between groups were larger in more generous welfare states. However, and importantly, in absolute terms the risk of social exclusion among disadvantaged groups was still far below the levels faced by the corresponding groups in less generous welfare states. In contradiction to the crowding-outhypothesis, welfare state generosity appeared to benefit all individuals in terms of overall lower levels of non-participation, both formal and informal. Our findings are thus in line with previous findings that a comprehensive welfare state is positively associated with social participation, [24,37].

Although it is not the main hypothesis of this paper, the results also demonstrated that welfare resources did not reduce the risk of social exclusion among disadvantaged groups also in relative terms. To the contrary, our analysis demonstrated that the most advantaged groups, in terms of good health, employment and high educational attainment, benefitted equally and in some instances more than disadvantaged groups in terms of informal social participation. These findings contradict recent studies that demonstrated that welfare generosity compensated disadvantaged individuals more [21,39]. One interpretation of these results is that individuals who are disadvantaged in health combined with other social disadvantages benefit the most from financial resources (benefits). Individuals who do not face these disadvantages in health and social position on the other hand, have sufficient financial resources, and thus profit more from certain services; for example, childcare services, freeing up additional time and opportunity to participate [10]. In sum, all social groups benefit from welfare generosity, but there are different mechanisms underlying the associations for different social groups. When the most advantaged individuals benefit more than the disadvantaged, the already strong inclination to participate in generous welfare states might be additionally boosted by the same mechanism.

### Strengths and limitations

While we outline a number of plausible theoretical pathways between welfare generosity and social participation, we are not able to separate these in empirical analysis. Nevertheless, we believe we are able to distinguish broadly between our two theoretical perspectives, the welfare resources perspective and the crowding-out theory. The results of this paper are in consistence with the former over the latter. Investigating the specific mechanisms underlying the observed pattern, although an important research agenda, is beyond the scope of this paper and would also imply the use of longitudinal individual level data. We do not, however, believe that the problem with consistency between theoretical model and empirical analysis is poorer than in most social science studies. In fact, it can be argued that the use of multilevel statistical methods which allows simultaneous investigation and control for both individual level and country level variables – and interactions between the two levels, as well as our operationalization of welfare generosity and focus on disadvantaged groups, *advance* on previous studies in terms of internal cohesion between theory and analysis.

There are however important limitations in this study. One of them are the mean VIF values, which were greater than 1 in all the analyses indicating a degree of multicollinearity (2.32 and 3.33, Tables 2(A) and 3(A) and 2.32 and 3.34, Tables 2(B) and 3(B) [53]. Given the fact that the analyses included interaction terms we conclude that multicollinearity was present, but did not represent a substantial problem.

Further, the relationships between welfare generosity and social participation might be influenced by confounding variables. To test the validity of our findings we have thus performed a series of sensitivity analyses.

Firstly, we included a measure for income inequality (GINI) and country wealth (GDP), one by one in each analysis. Neither income inequality nor country wealth was significantly associated with non-participation in informal networks. For both analyses of non-participation in formal networks the results demonstrated a significant, although weak (B = -0.00004), negative association with country wealth. The association with welfare generosity was no longer significant. However, there was a high level of multicollinearity in these analyses, as the correlation between the GDP measure and welfare generosity was very high (Pearson’s *r* = 0.94). Thus we cannot separate the effect of these two variables on non-participation in formal networks (results available at request).

Ideally we would control for a number of level 2 variables. Due to the low number of countries we lack statistical power to include several contextual variables simultaneously in the same analyses. We also lack available comparable contextual data to control for distinct cultural and/or historical factors. We however inspected country-level residuals and performed sensitivity analyses by leaving out the most outlying countries. The main results did not substantially change, thus confirming the findings of this paper (results available at request).

Additionally, we performed analyses to check whether the effect of the combinations poor health and non-employment in addition to poor health and low education on non-participation in formal and informal networks, respectively, varied across countries (random slope). These analyses demonstrated that only the effect of the combination poor health and non-employment on non-participation in informal networks proved significant (results available on request). The insignificant results might be related to lack of statistical power to fit random slopes, as we only have 21 level 2 units.

To investigate the effects of the individual variables on social participation we also analysed the associations between respectively non-employment, low education and poor health in interaction with welfare generosity on non-participation. The analyses did not contradict the results presented (results available upon request).

Because the aim of this paper was to study the risk of social exclusion we found it theoretically most appropriate to dichotomise the dependent variables despite the loss of information. We however included an ordinal version of informal participation with responses ranging from 1–7 (low-high), and a scaled variable for formal participation, in multilevel linear regression analyses (xtmixed). Although some results were not statistically significant within conventional levels, the associations were in the same direction as in the logistic regression analyses. We therefore conclude that this sensitivity analysis supports our main findings. Lastly, although the European Social Survey is an academically driven survey aiming at high methodological standards and optimal comparability of the data collected with a target response rate at 70%, the actual achieved response rates of ESS round 3 (2006/7) differ between countries [46,54].

Summing up, the findings in this paper must be interpreted with caution. Despite the above mentioned limitations, the findings nonetheless contribute important insights into the way welfare generosity may moderate the risk of social exclusion in terms of non-participation, associated with poor health combined with other social disadvantages. The results show that there is no indication of a crowding-out tendency among disadvantaged groups in more generous welfare states. On the contrary, resources made available by the welfare state seem to matter to all individuals in terms of overall lower levels of non-participation. These results demonstrate the importance of linking health related social exclusion to the social policy context.

## Competing interests

The authors declare that they have no competing interests.

## Authors’ contributions

ED and TS conceived the research question and planned the initial design. KW contributed substantially in the development of the research question, conceptualization and theoretical approach, and interpretation of the results. TS facilitated the data and performed the statistical analysis. TS and KW drafted the manuscript. TS revised the manuscript with the assistance from KW and ED. All authors read and approved the final manuscript.
